# New law on HIV testing in Botswana: The implications for healthcare professionals

**DOI:** 10.4102/sajhivmed.v16i1.337

**Published:** 2015-09-11

**Authors:** Rofiah O. Sarumi, Ann E. Strode

**Affiliations:** 1 College of Law and Management Studies, University of KwaZulu-Natal, South Africa; 2 School of Law, University of KwaZulu-Natal, South Africa

## Abstract

**Background:**

Botswana is one of the countries with the highest HIV prevalence rates in the world. Innovative HIV testing strategies are required to ensure that those infected or at risk of infection become aware of their HIV status and are able to access treatment, care and support. Despite this public health imperative, HIV testing strategies in Botswana will in future be based around the principles in the new *Public Health Act* (2013). The present article describes the HIV testing norms in the Act, and sets out the strengths and weaknesses of this approach and its implications for healthcare professionals in Botswana.

**Objectives:**

To compare international norms on HIV testing with the provisions governing such testing in the new Botswana *Public Health Act* and to assess the extent to which the new Act meets international human rights norms on HIV testing.

**Method:**

A ‘desktop’ review of international human rights norms and those in the Botswana* Public Health Act*.

**Conclusion:**

HIV testing norms in the new *Public Health Act* in Botswana violate individual rights and will place healthcare workers in a position where they will have to elect between acting lawfully or ethically. Law reform is required in order to ensure that HIV testing achieves the joint goals of public health and human rights.

## Introduction

Botswana continues to have one of the highest HIV prevalence rates in the world.^1^ Although the rate of new HIV infections has dropped, the prevalence rate remains high amongst certain populations, such as young persons with an estimated 23% of 15–49 year-olds being HIV infected.^1^ In this context, increasing access to HIV testing as the gateway to HIV prevention and treatment is important, and international best practice requires innovative HIV testing strategies to reach those at risk.^2^ It is against this background that the recently introduced *Public Health Act* (2013) which deals directly with HIV testing services in Botswana should be reviewed.^3^ The present article maintains that the approach adopted by the *Public Health Act* does not follow a rights-based approach to accessing HIV testing as set out in international norms. The article describes some of the implications this approach has for healthcare workers (HCWs), and it concludes with recommendations for law reform.

## HIV testing: The human rights framework

A rights-based approach has been defined as ‘a conceptual framework for the process of protecting human rights, based on international human rights standards and operationally directed towards promoting and protecting human rights’.^4^ This human rights approach is reflected in the well-established HIV testing norms at an international level. Although these standards have evolved over time, reflecting changing public health approaches, they have continued to be based on the fundamental human rights to exercise one’s autonomy, to privacy and to access the highest attainable standard of healthcare.^5,6^

Early guidance was established in the HIV and Human Rights Guidelines, a set of international norms describing the way in which governments ought to respond to the epidemic.^7^ Issued jointly by the United Nations Programme on HIV/AIDS (UNAIDS) and the United Nations Office of the High Commissioner for Human Rights in 1996, the guidelines provide that governments should review and reform their public health laws to ensure that they protect the right to consent, privacy and confidentiality during HIV testing.^7^ In 2004, further guidance from UNAIDS and the World Health Organization (WHO) established the principle of a rights-based approach to HIV testing^3^ by stating that the only form of acceptable mandatory screening is that done on donated blood.^8^ It provided further that the ‘3 Cs’ (consent, counselling and confidentiality) should form the bedrock of HIV testing services.^3^ In this approach, the focus is on patients voluntarily electing to test for their HIV status.^8^

In 2007, there was a shift in the international guidance when UNAIDS and the WHO issued guidelines on Provider Initiated Counselling and Testing (PICT).^9^ These proposed an approach in which HIV testing was to be recommended to all patients who present themselves at a healthcare facility with certain conditions.^9^ If the offer of HIV testing was accepted, consent would be obtained for the test with the overriding principle being the best interests of the individual patient.^10^ This approach requires the giving to individuals of sufficient information to make an informed and voluntary decision to be tested, maintaining patient confidentiality, performing post-test counselling and making referrals to appropriate services.^10^ This shift was prompted by the new human rights goals of universal access to prevention, treatment, care and support services.^10^

## The new legal framework regulating HIV testing in Botswana

The *Public Health Act* (No. 23 of 2013)^3^ attempts to comprehensively address key public health concerns in Botswana by creating regulatory structures and setting normative standards on certain issues such as which diseases should be notifiable.^3^ Part XII of the Act identifies HIV as a significant public health issue facing Botswana, and it sets a number of norms relating to HIV prevention and control.^3^ These include seven norms for HIV testing, as follows.

### Access to efficient HIV testing services

The *Public Health Act* provides that there is an obligation on the Minister of Health to ensure that confidential HIV testing facilities are available to all persons over the age of 16 (section 104^3^). Furthermore, the services ought to be efficient as every person has a right to receive their HIV test results as soon as they are approved (section 111^3^).

### Consent must be provided for the testing

HIV testing may only be undertaken with the consent of the person or their parent (if they are under 16) (s 105) unless the test falls into one of the mandatory testing categories described below.^3^

### Mandatory HIV testing

Nonconsensual testing may be done in six situations: (1) if a mentally disabled person is incapable of providing consent, they may be tested without consent (section 105[c]),^3^ (2) if the HIV test is required under this or any other act, for example the compulsory HIV testing of any person convicted of rape or defilement under the Penal Code (section 108),^3^ (3) if the person is unconscious and unable to give consent,^5^ (4) where the medical practitioner believes that such a test is clinically necessary or desirable in the interests of that person (section 105[2]),^3^ (5) all donated blood and tissue, (s 106–107) and (6) before any dental or surgical procedure (section 109). If a patient refuses to consent, the HCW may carry out the test without consent or refer the person to another HCW to do the procedure (section 109).^3^

A HCW who conducts an HIV test without consent is indemnified against any civil or criminal liability that may arise out of the nonconsensual HIV testing (section 105[3]).^3^

### Pretest information

Pretest information should be provided to any person who is to undergo an HIV test (section 110).^3^

### Confidentiality of HIV test results

Users of test services are entitled to confidentiality regarding both their test results and information on their sexual behaviour or the use of drugs.^3^ Furthermore, the *Public Health Act* provides that all positive results must be confidentially recorded by HCWs in terms of the notifiable disease obligations (section 114).^3^ Such information may only be disclosed with consent (section 115) or in terms of the circumstances described below.^3^

### Nonconsensual disclosure of a person’s HIV status

HCWs may disclose a person’s HIV status without consent in three circumstances: (1) to a sexual contact or caregiver if after a reasonable period they have not made such a disclosure themselves (section 116[7]).^3^ (2) after the death of the person (section 115)^3^ and (3) where there may be disclosure to other HCWs directly involved in the care of the patient.^3^

### HIV testing may only be undertaken at designated HIV testing centres

Section 119 provides that HIV testing may only be undertaken at a designated HIV testing centre.^3^

## Review of the new legal framework for HIV testing in Botswana

The provisions in the new *Public Health Act* strengthen patient rights by providing, firstly, that there is a positive obligation on the state to provide confidential HIV testing facilities to all persons over the age of 16.^3^ Given that HIV testing is the gateway to both HIV prevention and treatment, the Act makes access to these services a fundamental human right. If the State fails to make such services available, it could be held accountable for its inaction. Secondly, the testing services must be provided in a manner that respects rights, in that consent must be obtained from patients and their rights to privacy protected.^3^ Furthermore, patients must be given information before the test and this promotes their rights to autonomy in the decision-making process. Thirdly, the service must also be efficiently provided as the test results should be made available to patients as soon as the result is obtained and approved, which promotes their right to the highest attainable standard of healthcare. Fourthly, in line with international norms, all donated blood and tissue must be tested for HIV. Fifthly, the Act lowers the age of consent to HIV testing to 16, so enabling young persons at risk of HIV infection to become aware of their HIV status independently.^5^ Previously this was not a legal right as the *Children’s Act* is silent on the issue^11^ but it was allowed in terms of the National Guidelines for HIV Testing and Counselling of 2009.^12^

Based on the above provisions, those in the new *Public Health Act* appear on the face of it to be in line with the ‘3Cs’ as required in terms of international guidance issued by the WHO and UNAIDS. The Act also promotes a number of fundamental human rights as it protects the rights to autonomy and privacy. However, all of these rights are undermined by claw-back clauses in the Act. Firstly, the right to voluntarily consent to all forms of HIV testing is severely limited by the seven forms of mandatory or compulsory HIV testing that may take place in terms of the Act. As stated above, only one form of mandatory testing is allowed in terms of international HIV testing norms – the testing of donated blood. However, the drafters of the Act have created a further six circumstances in which testing may be undertaken without consent. It is particularly concerning that all persons undergoing surgical and dental procedures must be tested for HIV.^3^ This means that all persons visiting healthcare services for routine dental check-ups or minor procedures such as the removal of an ingrown toenail will be subjected to mandatory HIV testing. The public health value of testing all persons before surgical or dental procedures is unclear, given the use of universal precautions. The personal benefit to the patient is also unclear as there is no direct obligation to provide post-test counselling or refer them for treatment. In addition to mandatory testing, the Act also allows HCWs to undertake HIV testing where they believe that the testing is clinically necessary or even simply ‘desirable’ in the interests of that person.^3^ This creates a very low threshold at which HCW paternalism could override patient autonomy, which undermines the right to autonomy as it places the decision to test in the hands of the HCW. This opens the door to *inter alia* HIV testing practices being driven by, for example, stigmatising or discriminatory attitudes towards certain populations such as men who have sex with men, or sex workers.

Secondly, the right to privacy established in the Act is undermined by the sweeping powers of HCWs to disclose the HIV status of a patient to any sexual contact or caregiver of the patient if they become aware that the patient has not made such a disclosure themselves.^3^ This power is out of step with international norms that would generally only require disclosure if the sexual partner or caregiver is at risk of HIV infection.^13^ Mandatory disclosure not only violates the right to privacy, but it also places persons living with HIV at increased risk of stigma and discrimination. Furthermore, the Kenyan High Court recently found that the term ‘sexual contact’ in the Kenyan *HIV/AIDS Prevention and Control Act* (No. 14 of 2006) violated the principle of legality in that it was ‘vague and overbroad and lacks certainty’.^14^ The court was of the opinion that HCWs would not be able to comply with the provision as it was not clear who would be considered a sexual contact.

Thirdly, although the Act allows children of 16 and above to consent independently to HIV testing, this is an acontextual approach as there is nothing in the laws of Botswana which allow children of this age to independently consent to HIV treatment. This means that children will still require parental assistance, which may act as a barrier to some of them accessing antiretrovirals.

Fourthly, the approach taken in the Act to mandatory testing and disclosure places HCWs in an ethical dilemma. The Medical Council (Professional Conduct) (Amendment) Regulations provide that doctors have an ethical obligation to maintain confidentiality and may only violate this rule in limited circumstances, such as when ordered to do so by a court.^15^ This means that doctors complying with the Act will be violating professional ethical obligations.

Finally, the narrow approach taken in the Act to limiting HIV testing services to designated facilities means that innovations such as home or self HIV testing cannot be rolled out in Botswana as they are expressly prohibited by law; this undermines the right to the highest attainable standard of healthcare.

## Implications of the new HIV testing provisions for healthcare workers in Botswana

There are several implications of this new law for healthcare professionals working in Botswana, including that they:

- need to be aware that they may be asked to act unethically but legally in carrying out mandatory HIV testing, particularly before all surgical and dental procedures. In this regard, it is recommended that practitioners consult with their professional structures to obtain advice on what to do in such instances
- will be under a legal obligation to disclose the HIV status of, for example, pregnant HIV-positive women if they are not convinced that the patient has made the disclosure herself to her partner. This may place women at risk of domestic violence or other negative consequences^12^
- may lawfully disclose a patient’s HIV status to other HCWs directly involved in the care of the patient
- will need to advise children over the age of 16 that, even if they consent on their own to an HIV test, they will need parental assistance to access HIV treatment
- will be unable to offer HIV testing to children under the age of 16 who do not have a parent or guardian to advise them as there is no provision in the Act for any alternative proxy consenter
- ought to provide pretest information but no legal obligation to provide post-test counselling
- cannot offer new innovations such as home HIV testing for these will be illegal as testing is limited to being done at authorised centres.

## Conclusion

Sadly, the new 2013 *Public Health* Act^3^ in Botswana goes against international best practices as laid out in instruments such as the UNAIDS/WHO Policy Statement on HIV,^8^ the HIV and Human Rights Guidelines,^7^ and the PICT guidelines.^9^ Although the Act provides a veneer of human rights, HIV testing will generally now be undertaken in a coercive manner, which undermines efforts to increase awareness of one’s HIV status. The drafters of the Act have also misunderstood the shift in international norms as, although there is a focus on increasing access to HIV testing, it still requires such testing to be done in a way that is consistent with human rights norms. The Botswana legislature has elected to ignore this approach. It is unlikely that the current coercive approach to HIV testing as set out in the *Public Health Act* will result in greater individual awareness of HIV status, as most of the testing will be directed at HIV testing in the interests of the healthcare provider.

### Recommendations

We submit that there is a need to reform the *Public Health Act* to ensure that HIV testing services are provided in a way that does not infringe people’s rights. As a minimum, the power to test patients without their informed consent should be removed and the mandatory disclosure provisions limited to situations where a third party is at significant risk of HIV infection.

## References

[1] UNAIDS HIV and AIDS estimates. (c2012). http://www.unaids.org/en/regionscountries/countries/botswana.

[2] Kalichman SC, Simbayi LC. HIV testing attitudes, AIDS stigma, and voluntary HIV counselling and testing in a black township in Cape Town, South Africa. Sex Transm Infect. 2003;79:442–447 PMID: 14663117, http://dx.doi.org/10.1136/sti.79.6.442 14663117 10.1136/sti.79.6.442 PMC1744787

[3] Government of Botswana Public Health Act. (2013). https://dl.dropboxusercontent.com/u/1576514/Botswana%20Public%20Health%20Bill%202012.pdf.

[4] Office of the United Nations High Commissioner for Human Rights. Frequently asked questions on a human rights-based approach to development cooperation. (c2006). http://www.ohchr.org/Documents/Publications/FAQen.pdf.

[5] UNO. International Covenant on Civil and Political Rights. Geneva: United Nations Organization; 1966.

[6] UNO. International Covenant on Economic, Social and Cultural Rights. Geneva: United Nations Organization; 1966.

[7] UNAIDS. (c2006). UNAIDS international guidelines on HIV/AIDS and human rights. Consolidated version..

[8] UNAIDS. (c2004). UNAIDS/WHO policy statement on HIV testing..

[9] WHO. (c2007). Guidance on provider-initiated HIV testing and counselling..

[10] Heywood MJ. The routine offer of HIV counselling and testing: A human right. HIV AIDS Policy Law Rev. 2006;11:71–72. PMID: 17375428. 17375428

[11] Government of Botswana. Children’s Act, 2009. (c2009). http://www.aclr.info/images/stories/uploader/Publication_files/Acts/Botswana_Children_Act_08_of_2009.pdf.

[12] Government of Botswana. National guidelines for HIV testing and counselling centres, 2009. (c2009). http://www.gov.bw/Global/MOH/PC_MOH_01.pdf.

[13] Cuerrier R v [1998]. Can. Sup. Ct. LEXIS 4312.

[14] AIDS law project v Attorney General of Kenya and others, Case no. 97 of 2010. Kenya Law. 2015.

[15] Government of Botswana. Medical Council (Professional Council) (Amendment) Regulations 77 of 1999. Gaborone: Government of Botswana; 1999.

